# Medical Practitioners’ Views on Barriers in Collaboration with Dentists in Long-Term Care Settings

**DOI:** 10.1177/23800844241305015

**Published:** 2025-02-10

**Authors:** S. Tada, S.M.Y. Koh, G.K.Y. Lee, M.L. Wong

**Affiliations:** 1 Faculty of Dentistry, National University of Singapore, Singapore

**Keywords:** oral health, geriatric dentistry, interprofessional relations, health services for the aged, health care disparity, long-term care

## Abstract

**Introduction:**

In long-term care settings (LTCs), oral health care often remains fragmented from other geriatric care services. Medical practitioners (MPs) typically take the lead in addressing medical aspects of geriatric care plans, making it essential for them to understand the importance of oral health and to collaborate with dental professionals. However, little is known about MPs’ perceptions toward oral health management in LTCs. This qualitative study aimed to gain an in-depth understanding of MPs’ views on oral health management in LTCs and explore challenges in collaborating with dental professionals in Singapore.

**Methods:**

Participants were recruited using a purposive sampling strategy, initially through targeted outreach to those with experience in LTCs, followed by snowball sampling to identify additional participants until data saturation was achieved. One-to-one interviews with participants were conducted via teleconferencing using a semi-structured interview guide. The transcripts were analyzed using a hybrid (inductive and deductive) thematic analysis supported by NVivo data management software.

**Results:**

Participants (n = 13) felt that oral health care was currently not well-integrated into the existing LTC system. They frequently encountered oral health issues but arranged for dental appointments only when their patients complained of acute symptoms. Key barriers identified were (1) a cultural misconception within the eldercare community that poor oral health was an inevitable part of aging, (2) systemic barriers related to the inadequate geriatric oral health care services and infrastructure, and (3) knowledge gaps in geriatric oral health management among MPs. These factors collectively hindered effective collaboration with dental professionals in LTCs.

**Conclusion:**

Participants emphasized the lack of oral health care integration in LTCs, identifying cultural, systemic, and internal barriers. Strengthening collaboration with dental professionals in LTCs, implementing oral health education for the eldercare community, and introducing domiciliary services could address these challenges and support more effective geriatric care, with insights for similar systems globally.

**Knowledge Transfer Statement:**

This qualitative study highlighted a critical gap in oral health care integration within eldercare described by the MPs in this study in Singapore’s long-term care settings. Although findings are context specific, they may offer insights for aging nations facing similar challenges. Overcoming misconceptions about aging and oral health, improving systems, and educating MPs are key to fostering interdisciplinary collaboration and enhancing eldercare. Addressing these barriers could improve the overall well-being of care-dependent older adults.

## Introduction

Globally, oral health care in long-term care settings (LTCs) tends to remain overlooked or fragmented within the general geriatric care agenda despite a vital need for a collaborative approach (Harnagea et al. 2017; WHO Ageing and Health 2021). As a result, access to oral health care service is limited for dependent older adults who face challenges in daily living activities and self-management of health, exposing them to the risk of oral health deterioration (Aida et al. 2022; Chávez et al. 2022). In fact, older adults in LTCs worldwide are prone to have a high prevalence of poor oral hygiene, untreated tooth decay, and ill-fitting and poorly maintained dentures (Thean et al. 2009; Janssens et al. 2017; Yoon et al. 2018). As emphasized by the WHO in their 2022 report (WHO 2022), oral health plays a pivotal role throughout a person’s life, affecting essential functions for daily living, social activities, and personal fulfillment. This underscores the importance of fostering stronger partnerships between dental professionals and other stakeholders in LTCs.

Extensive evidence underlines the interconnectedness of oral health with overall health; several medical conditions can affect oral health or be influenced by it among dependent older adults. For example, their mental and physical functional decline can lead to poor oral hygiene and subsequent oral and dental issues (Lauritano et al. 2019). Polypharmacy, which is the most prevalent among vulnerable older adults, can cause dry mouth, which increases the risk of oral infections (Thomson et al. 2021). In turn, increased oral bacteria can worsen respiratory conditions (Scannapieco et al. 2022), and severe chronic periodontal inflammation can complicate the management of diabetes (Kuo et al. 2008). Declined oral function can affect the ability to eat properly, potentially leading to malnutrition and frailty (Iwasaki et al. 2021). Therefore, it is crucial for health care providers in LTCs to be aware of these interconnections and the vicious cycle between overall health and oral health, working collaboratively with dental professionals to address both medical and oral health needs.

Currently, in LTCs, medical practitioners (MPs) often take the lead in addressing the medical aspects of geriatric care plans. It is therefore crucial that they understand the clinical significance of oral health management for older adults and collaborate with dental professionals (Kossioni et al. 2018). However, few studies, primarily from Western countries, have explored MPs’ attitudes and approaches to oral health management. These reports suggest that MPs often lack awareness of geriatric oral health and have limited knowledge in managing oral health issues in older patients (Andersson et al. 2007; Appleby et al. 2016). This situation may be mirrored in other aging countries, but little is known about MPs’ perceptions regarding collaboration with dental professionals in caring for the oral health of older adults in LTCs.

In Southeast Asia, Singapore is one of the most rapidly aging nations, now facing a rising burden of the dependent older population. The old-age support ratio (the ratio of the working-age population aged 20 to 64 y per person aged 65 y and older) of the population has declined from 10.9 in 1990 to 3.8 in 2022 (Department of Statistics, Ministry of Trade and Industry, Republic of Singapore 2021). It is projected that 1 in 4 citizens (23.8%) will be aged 65 y and older by 2030, rising to almost 1 in 2 by 2050 (Association of Southeast Asian Nations 2021). About 80% of the total disease burden in Singapore is due to noncommunicable diseases (GBD 2019; Demographics Collaborators 2020), and the proportions of those with difficulty performing at least 1 basic daily activity were 16.2% among those aged 75 to 85 y and 40.8% among those aged 85 y and older in 2020 (Department of Statistics, Ministry of Trade and Industry, Republic of Singapore 2021). Corresponding with this swift demographic shift, the government has been conducting a full-fledged reform of the health care system. These efforts have significantly enhanced health care capacity, affordability, and financial protection for the older population. The key features of Singapore’s health care system, including its approach to LTCs, have been described previously (Tan et al. 2021).

In Singapore, LTC services refer to home-based care service or nursing home care service, with a range of services designed to meet the medical, personal, and social needs of individuals who are unable to fully perform basic activities of daily living over an extended period. In particular, home-based health care services are designed to assist home-bound older adults who face difficulties accessing geriatric care services in the community. In contrast, nursing homes are intended for dependent older adults in need of specialized care and assistance, which extends beyond the scope of home care services or community-based settings. These LTC medical services are supported by a combination of public insurance schemes and government subsidies, ensuring that care-dependent older adults can access necessary medical care without prohibitive costs. Within Singapore’s LTC sector, medical doctors routinely visit patients to address their medical needs. Most of these doctors are general practitioners with family medicine training, either through practice or a degree/diploma program, known as family physicians, or they are registered specialists in geriatric medicine. As of 2021, of the total 16,044 registered MPs, 2,312 were registered as family physicians and 147 as geriatric medicine specialists (Singapore Medical Council 2021).

Despite the well-developed LTC system in Singapore, professional dental services are not integrated in the existing LTC service framework.(Tada, Lee, and Tay 2023) Most nursing home residents and homebound older adults must travel to off-site public or private dental clinics, often facing challenges such as transportation, scheduling difficulties, and financial barriers. In response, volunteer dentists provide free on-site screenings and basic treatments in nursing homes; however, these efforts remain scarce and ad hoc due to limited resources. This highlights the need to better understand the challenges in achieving oral health care integration within LTCs, so that these barriers can be adequately addressed (Tada, Lee, Koh, et al. 2023).

Hence, this qualitative study aimed to provide an in-depth understanding of MPs’ views on oral health care partnership in LTCs in Singapore. Specifically, we aimed to explore (1) MPs’ awareness of oral health among dependent older adults, (2) MPs’ approaches to oral health issues faced by dependent older adults, and (3) MPs’ challenges when partnering with dental professionals in LTCs.

## Materials and Methods

The study was conducted in accordance with the Declaration of Helsinki and approved by the Institutional Review Board of the National University of Singapore (NUS-IRB-2020-202).

A qualitative descriptive design was used, grounded in an interpretivist paradigm, to explore the nuanced views of MPs on oral health care in LTCs. Through one-to-one semi-structured interviews, the aim was to capture the richness of their perspectives on oral health care for dependent older adults, their collaboration with dental professionals, and potential improvements in service provision. The interpretivist paradigm aligns with the goal to contextualize and interpret these narratives, acknowledging the subjective experiences of our participants and the influence of their social and professional environments.

To minimize researcher bias, 2 researchers (S.T. and S.M.Y.K.), who were unfamiliar with LTC clinical settings in Singapore and who did not have personal connections with the MPs, carried out the interviews. S.T. is a dentist who has the academic and clinical background of geriatric and primary care dentistry in Japan, whereas S.M.Y.K. has the academic background of social science. G.K.Y.L. also has the academic and clinical background of geriatric and primary care dentistry in Singapore and is familiar with the situation in LTCs in Singapore on an empirical basis. All of the research members are familiar with this research topic and have experience in qualitative research. Given S.T,’s background in geriatric dentistry, there was an initial presumption that oral health care would be underprioritized in LTCs. S.M.Y.K.’s social science background offered a complementary perspective on the social dynamics in these settings.

Participants were MPs who (1) were registered with the Singapore Medical Council as a medical doctor, (2) had working experience in LTCs (nursing homes and/or home medical care) in Singapore for at least 2 y, and (3) were able to communicate in English verbally and in writing. Purposive snowball sampling was used, starting with participants introduced by dentists with experience in LTCs. The first potential participants were MPs who met the inclusion criteria and who were introduced by dentists who had clinical experience in LTCs and had participated in another of the author’s research project. Once initial participants (*n* = 2) were interviewed, they were asked to nominate/introduce other potential MPs who might be interested in the study. Snowball sampling continued with data gathering and analysis occurring concurrently until no new themes or insights were identified from the interviews, indicating that additional data collection was unlikely to provide further information relevant to the study’s research questions (data saturation).

To enhance the diversity of views, participants were invited to recommend individuals from different workplaces, with varying experiences, genders, or ages. This approach helped us ensure that a broad range of experiences and perspectives were represented in our sample. Participation in the study was voluntary, and written informed consent was obtained from all participants.

Data collection for this qualitative study was conducted through one-on-one interviews via a video-conferencing platform (Zoom software, Zoom Video Communications), ranging from 30 min to 1 h each, between October and November 2021. This approach was chosen to allow participants to choose not to show their faces, fostering a more open and honest discussion of their personal experiences. Two trained researchers (S.M.Y.K. and S.T.) facilitated the interviews using a semi-structured interview guide (Tada et al. 2024). The interview guide was developed based on a literature review around the larger research questions of this study: (1) knowledge of current oral health care for older adults in LTCs, (2) collaboration with dental professionals, and (3) potential solutions to improve oral health care provision for older adults and collaboration with dental professionals. This guide was also tailored to encompass specific questions pertinent to the Singaporean context, thereby ensuring the relevance and applicability of our findings. The interview questions addressed their current knowledge of oral health care for older adults as well as their thoughts on the future of interdisciplinary eldercare teams for LTCs (Table 1). Examples of Singapore-specific questions included (1) case management of the oral health problem (“Could you share how you actually manage the cases when you do see oral health problems?” This question aimed to understand the specific actions and strategies MPs use to address oral health issues in the absence of integrated dental services in Singapore.) and (2) interaction with volunteer dentists in nursing homes (“For the nursing home that you are currently working with, do they have volunteer dentists coming down? How do you communicate with them?” These questions were tailored to reflect the unique aspects of the Singaporean LTC context, where dental services are not fully integrated and volunteer dentists play a crucial role in addressing oral health needs.).

**Table 1.** Overview of the Interview Guide Used in This Study.

Clinical experience in nursing homes	• What is your experience in providing medical care for the dependent elderly?
Current oral health of dependent older adults	• What is your understanding of the current situation of oral health care for the elderly in nursing homes?• What do you know about/what is your take on oral health care service in nursing homes in Singapore?
Relationship between general health and oral health of dependent older adults	• What do you know about the relationship between general health and oral health among the elderly?
Barriers and solution(management of oral health problems)	• Have you encountered any oral health problems among the elderly? How did you manage these cases?• When considering the management of malnutrition/aspiratory pneumonia, do you also consider the elderly’s oral health status?
Barriers and solution (geriatric education)	• Is dental education covered in the medical undergraduate curriculum?• Are training and education programs on geriatric oral health care readily available for medical professionals?• Do you think there should be more overlap between the medical and dental disciplines?
Barriers and solution (interdisciplinary collaboration)	• Were there instances in which you were required to communicate with dental professionals that provided dental checkups and treatment for the elderly in nursing homes? • What were the nature of these interactions/what do these interactions usually involve?• Were there instances in which you were asked by dental professionals to provide medical advice? • What do these interactions usually involve (e.g., prescription of medication, medical clearance for dental procedures)?• What can be done to improve the working relationships/collaborations with dental professionals?

To ensure the rigor and trustworthiness of the study, the following strategies were used. First, triangulation (Patton 1999) was applied by incorporating multiple data sources and involving several investigators with different backgrounds. Data were collected from a diverse group of MPs across various LTC settings, ensuring a broad range of perspectives. Diversity among researchers also provided independent insights and facilitated cross-checking interpretations, thereby enhancing the credibility and robustness of our findings. Second, throughout the research process, the research team, comprising members from various backgrounds, engaged in regular discussions. These discussions were crucial for surfacing and challenging assumptions, ensuring that the research questions and data interpretations were critically examined and not unduly influenced by individual biases. This iterative dialogue contributed to a more balanced and comprehensive understanding of the data.

All interviews were recorded for later verbatim transcription and analysis. The interviews were audio recorded and the transcripts managed using NVivo 12 (QSR International Pty. Ltd). The transcripts were analyzed by 3 research members (S.T., S.M.Y.K, G.K.Y.L.) to maintain the high accuracy of these transcripts. A hybrid approach to thematic analysis was used, combining an inductive process driven by the data with a deductive framework guided by a codebook developed through a literature review (Fereday and Muir-Cochrane 2006). The details of the analysis process are shown in Table 2.

**Table 2.** Thematic Analysis Process.

Stage	Details	Analyzing Device
Coding template creation	One researcher (S.M.Y.K.^1^) created a coding template based on the interview guide.	Manual
Familiarization	To familiarize themselves with the content of the data sets, 3 researchers (S.M.Y.K., S.T., G.K.Y.L.) individually listened to the audio-recorded data and read the transcriptions at least 5 times.	Manual NVivo (transcription)
Coding	• Deductive approach: Two researchers (S.M.Y.K., S.T.) independently coded the data referring to the prepared coding template. • Inductive approach: When there were no templated codes representing certain contents in the data, new codes were created. After this individual coding process, S.M.Y.K. checked if there were any discrepancies between the researchers. If there were, the researchers discussed and found mutually agreeable codes.	Manual
Generating initial themes	The initial codes were jointly reviewed by S.M.Y.K. and S.T., and they identified patterns among the codes and named them as themes. After this initial theme generation, S.M.Y.K. input all the initial analysis into NVivo software to create the list of the initial codes, themes, definitions, and relevant references.	Manual NVivo (analysis)
Reviewing codes and themes	The project leader (S.T.) reviewed and identified salient codes and themes. S.T. raised any codes and themes that were unclear and further discussed and recategorized these with other members (S.M.Y.K. and G.K.Y.L.).	NVivo (analysis)
Defining and naming themes	Based on the final list of codes and themes, the three researchers (S.T., S.M.Y.K., G.K.Y.L.) reviewed these together and refined the definition and name of each code and theme to make them more succinct and accurate.	Manual

## Results

Fourteen MPs agreed to participate in the study; however, 1 MP withdrew due to schedule conflicts (Table 3). In total, 13 MPs (8 males and 5 females, 41 [interquartile range: 39 to 52] y old) participated in the interview. The number of years of clinical experience in LTCs including both home-based and nursing home settings as a MP ranged from 3 to 20 y (median 9 y). Most participants served in LTCs including home medical care and/or nursing homes as a full-time position, holding a qualification of advanced medical training in area such as family medicine, geriatric medicine, and mental health (dementia). All MPs belonged to different institutions, and only 3 MPs in this study had a partnership with dental professionals in their working institutions.

**Table 3.** Demographic Characteristics of Each Participant.

Index No.	Gender	Age	Attainment of Advanced Medical Training in Primary Care and Geriatrics	Clinical Experience in Long-Term Care Settings			Partnership with Dental Professional in Their Institution
				Experience of Home Medical Care	Experience of Nursing Homes	Years of Experience	
MP01	Withdrew from interview due to scheduling conflicts						
MP02	Male (M)	51	Yes (Family physician)	Yes	No	9	No
MP03	Female (F)	39	Yes (Family physician)	Yes	Yes	6	No
MP04	M	39	Yes (Family physician)	Yes	Yes	5	Yes
MP05	M	39	Yes (Family physician)	Yes	Yes	5	No
MP06	F	34	No (General practitioner)	Yes	Little	3	No
MP07	M	40	Yes (Family physician with geriatric medicine diploma)	No	Yes	6	Yes
MP08	F	45	No (General practitioner)	Yes	Yes	12	No
MP09	F	39	Yes (General practitioner with geriatric medicine + family medicine diploma)	No	Yes	8	No
MP10	M	53	Yes (Family physician)	Yes	Yes	20	Yes
MP11	M	53	Yes (Geriatrician)	No	Yes	20	No
MP12	M	41	Yes (General practitioner under resident training in homecare)	Yes	Yes	10	No
MP13	F	41	Yes (General practitioner with mental health diploma)	Yes	Yes	10	Yes
MP14	M	53	Yes (Family physician)	Yes (main)	Yes (consultation)	20	No

## Oral Health Awareness

Overall, participants acknowledged that older adults in LTCs in Singapore generally had poor oral health, which requires professional dental intervention.

Poor oral health is quite common actually. (MP02, Family physician, 51 y)

You see the condition of the teeth . . . you can be quite shocked, actually. (MP04, Family physician, 39 y)

I would say, all of the residents have dental needs, just as everyone actually has dental needs. The elderly are no less that and in fact even more important for them to receive dental care. (MP07, Family physician with geriatric medicine diploma, 40 y)

The participants appreciated the impact of oral health on general health such as older adults’ well-being, nutrition, and respiratory infection (Table 4).

When we do a basic oral toilet for them at home, they just feel better. They look better, they’re more comfortable. They don’t look so miserable in a bed. (MP08, General practitioner, 45 y)

This is very important because oral health is closely associated with their general state of health, especially nutrition. (MP11, Geriatrician, 53 y)

The concern is also that they might get pneumonia because of the poor oral hygiene. So, I do think it’s very important. (MP09, General practitioner with geriatric medicine and family medicine diploma, 39 y)

**Table 4.** Oral Health Knowledge Noted by the Participating Medical Practitioners (MPs).

Topic			Definition	No. of MPs	
1	Oral health and well-being	Oral health and physical well-being	MPs noted that poor oral health will result in discomfort and pain for seniors	10/13	13/13
		Oral health and mental well-being	MPs noted that poor oral health will affect their self-image, self-esteem, emotion, mood, and life satisfaction	10/13	
		Oral health and social interactions and communication	MPs noted that poor oral health may affect their motivation to interact with others and may cause them to have difficulty articulating and speaking	4/13	
2	Oral health and malnutrition		MPs noted that poor oral health will affect their ability to chew and eat, which will affect their nutrition	13/13	
3	Oral health and aspiration pneumonia		MPs noted that poor oral health may lead to development of aspiration pneumonia	9/13	
4	Oral health and endocardial disease/heart disease		MPs noted that oral bacteria may cause inflammation and infection in the heart or the arteries	4/13	
5	Oral health and osteoporosis		MPs noted that medication for osteoporosis is related to osteonecrosis of the jaw (MRONJ)	5/13	
6	Oral health and polypharmacy		MPs noted that polypharmacy may contribute to the occurrence of dry mouth (xerostomia)	2/13	

## Approach to Oral Health

### Contrasting Response to Oral Health Issues: Proactive versus Passive

Despite having a broad awareness of oral health, there were 2 types of responses to oral health issues among the participating MPs. One group included those who emphasized the crucial role of oral health and proper oral health care for dependent older adults, recognizing its profound impact on their overall health and well-being. This group proactively incorporated dental and oral health assessments into their medical reviews and/or educated caregivers about the importance of oral hygiene management to prevent oral issues.

Basically, I see the number of teeth, I see the cleanliness of the teeth, whether there’s any plaque or things like that, any ulcers, the moistness of the buccal cavity. . . . We will have to explain to them that good oral hygiene actually reduces the risk of aspiration pneumonia. (MP09, General practitioner with geriatric medicine and family medicine diploma, 39 y)

If the caregiver has not been doing any oral hygiene at all we will administer some basic advice on how to do simple brushing with sponges or sodium bicarbonate powder or chlorhexidine mouthwash. (MP05, Family physician, 39 y)

The other group felt that oral health care fell outside of the purview of medical care. They did not have a clear clinical sense of the link between poor oral health and general health such as aspiration pneumonia or eating disability of older adults.

I would say that unfortunately, this is not really our main focus . . . a lot of the times we look into the mouth really to look for more of the medical aspect. (MP06, General practitioner, 34 y)

The thing is, somehow dental intervention doesn’t quite reverse the bad dentition . . . maybe even the most important aspect of quality of life. (MP10, Family physician, 53 y)

This group did not mention that they proactively checked the oral and dental health of older adults as a part of their routine practice. Instead, their examinations were focused on medical issues such as oral thrush and dry mouth or were conducted in response only to acute oral symptoms reported by older adults or their caregivers.

### Reactive Practice to Oral Health Issues

Participants commonly came across the following oral health issues among older adults in LTCs: (1) eating problems due to ill-fitting dentures, (2) poor dental hygiene, (3) acute pain due to dental caries, (4) loose teeth to be extracted, (5) dry mouth, and (6) dysphagia.

However, regardless of their level of awareness or attitude toward oral health, none of the participants regularly or frequently engaged in direct interactions with dentists to discuss oral health issues faced by the older patients.

If you’re talking about like meeting dentists face to face or having a consultation with them in any way . . . then NO.” (MP05, Family physician, 39 y)

With regard to impaired eating function, they typically consulted with dietitians or speech therapists and made recommendations to soften or puree food or prescribe nutritional supplement drink, rather than consulting with dentists about oral rehabilitation. Consequently, professional dental intervention was not actively initiated by them.

Most times when I do refer, it’s usually to speech therapists . . . if there’s other oral issues, they [nursing homes] will have already existing in-house speech therapy appointment. (MP02, Family physician, 51 y)

The general culture is that we usually use like a milk supplement to augment their nutrition. . . . So rather than really tackling the root cause, which might be poor dental health, we usually prescribe them milk supplements. (MP06, General practitioner, 34 y)

Participants did not encourage older adults to seek professional dental care proactively. Referrals to dentists were made only when acute symptoms were present, such as severe pain, abscess, and/or loose teeth to be extracted. In the case of mild gingival inflammation or dry mouth, MPs managed to treat these patients by prescribing medications with their own medical judgment.

We don’t actually push [older adults] very hard [to see a dentist] . . . sad to say.” (MP02, Family physician, 51 y)

### Barriers Hindering MPs from Collaborating with Dental Professionals

There were several obstacles that participating MPs face in partnering with dental professionals. These barriers are categorized into cultural, systemic, and internal barriers. The concept map illustrating the interrelationship and impact of barriers on their awareness and approaches is shown in the Figure.

**Figure.**
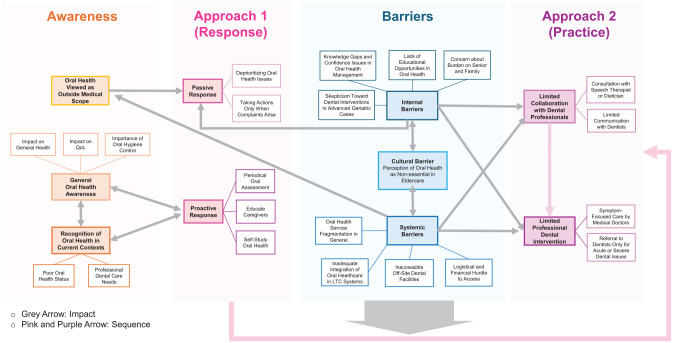


#### Cultural misconception about oral health as nonessential for eldercare

The participants were aware that there was a pervasive belief within the eldercare community, families, and even among medical professionals that poor oral health is “part of the ageing process,” resulting in lower priority in elderly care.

I think many times a lot of the patients themselves or even the families and maybe ourselves just assume that as they age, their dentition will decline and obviously they won’t be able to chew that well. (MP12, General practitioner under resident training in homecare, 41 y)

I guess this [oral health] is really very much neglected. Not just in nursing home settings but in the hospital and in the home settings by caregivers. (MP11, Geriatrician, 53 y)

The participants shared a dilemma that family members were reluctant to pursue professional dental treatment, even when the participants, as MPs, attempted to convince them that it may bring better comfort and oral function for their older adults.

Their family members are also not aware of the need. So even if you tell them to go and see a dentist, go and do this and do that, they will tell you, “Why must see a dentist?” (MP13, Geriatrician, 41 y)

In addition, some participants with greater awareness expressed concerns about the general lack of such awareness among medical doctors, which could result in unnoticed dental issues for dependent older adults who are unable to communicate their problems effectively.

If there’s no awareness, then like I said, I think some physicians or even some other care professional may not even really go through the oral screening. (MP02, Family physician, 51 y)

#### Systemic barriers

Participants consistently raised concerns regarding the inadequacy of geriatric oral health care services and the associated infrastructure. Several key issues have been discussed as common points of contention, hindering collaboration between MPs and dental professionals to co-manage the overall well-being of dependent older adults. These issues include:

1. Systemic fragmentation of oral health from other geriatric areas: The participants felt that oral health service had been fragmented from the existing geriatric care system. This separation led them to view these issues as outside their purview, making them hesitant to refer patients to dental professionals

I feel that the dental setup is always very separate from the medical setup . . . so it makes it harder for people to see it as being “oh, we are actually working towards the same thing,” you know, in that sense. (MP13, Geriatrician, 41 y)

2. Inadequate integration of oral health care in LTC systems: Participants emphasized the lack of a structured and systematic framework to incorporate oral health screening and professional care into LTC systems for elderly populations, which hinders the partnership with dental professionals.

There’s no real structure in screening for dental health in this population. . . . You certainly don’t have anything for nursing homes. (MP04, Family Physician, 39 years)

In the nursing home setting, in fact . . . it is not easy to get a dentist to come into the nursing home to see the residents on an ad hoc basis. (MP11, Geriatrician, 53 years)

3. Inaccessible off-site geriatric dental facilities: The participants were reluctant to refer older adults in LTCs to public dental clinics geriatric dental care due to long waiting times.

For those nursing home which has no dentist, we have to either refer them to the hospitals, either through the polyclinic. . . . So they join that long queue . . . the waiting times are, unfortunately long, not short. (MP07, Family physician with geriatric medicine diploma, 40 y)

In addition, they were also uncertain if private dental clinics were a viable alternative, given doubts about the clinics’ and dentists’ ability to manage geriatric or bedbound patients effectively.

I’m not sure if the normal dental clinics in the neighborhood will be able to handle bedbound patients. (MP12, General practitioner under resident training in homecare, 41 y)

The nursing homes might not have the resources to bring these nursing home residents to the [private] dental clinics. Yeah, so I have been stuck on a few occasions because of tooth aches. (MP08, General practitioner, 45 y)

4. Logistical and financial barriers to access to professional dental service: The participants were also hesitant about referring older adults in LTCs to off-site dental clinics or hospitals due to issues with affordable transportation and financial constraints, complicating access to timely dental treatment.

The main issue they have is mobility. So, to bring them out to a dental clinic for any kind of assessment would be quite a hassle; a lot of them will need special transport. And if you talk about that, the costs of transport will come in, that's one thing. (MP14, Family Physician, 53 years)

I think dental treatment generally, peoples’ idea of it is that it’s quite costly. Yeah. And I don’t think that there’s that much subsidy as compared to medical. (MP06, General Practitioner, 34 years)

#### Internal barriers

The participants also raised factors within the medical community that hindered the partnership with dental professionals. These internal barriers reflected a complex interplay of educational shortcomings, skepticism, and practical concerns, all of which hindered the partnership with dental professionals.

1. Lack of educational opportunities in oral health: The participants had limited or no exposure to oral health topics within their medical education. This lack of formal education means that oral health is often not prioritized or fully understood.

This [oral health] is not something that is taught very much in medical school. (MP09, General Practitioner with Geriatric Medicine + Family Medicine Diploma, 39 years)

All of these things, to be very honest, as a medical professional, I was never taught these things. Never, ever . . . only when I got to know this particular dentist, really opened my eyes to this. (MP13, Geriatrician, 41 y)

2. Knowledge gaps and confidence issues in oral health management: Due to the absence of formal training opportunities in this area, even the more knowledgeable participants lacked confidence in their ability to assess and manage oral health; this lack of confidence made it challenging for them to determine which cases would benefit the most from dental consultation or intervention.

I really have no idea on how to manage the dental conditions that many of them have. (MP05, Family physician, 39 y)

If you don’t assess that, you’re not going to pick that up and if you don’t pick that up then you’re not going to have the opportunity to create a structure for collaboration. (MP07, Family physician with geriatric medicine diploma, 40 y)

3. Skepticism toward dental interventions in advanced geriatric cases: Participants with lower oral awareness shared doubts and uncertainties regarding the clinical benefits and outcomes of dental interventions for elderly patients with complex health conditions.

I’m not sure . . . what is . . . how much additional benefit, you know, like which patient group will get the most benefit out of a consult with the dental professional? (MP03, Family physician, 39 y)

The majority are not eating well because of the comorbidities . . . whereas for dental, I’ll say a small proportion. There’s a small proportion who might have been issues affecting their intake. (MP14, Family physician, 53 y)

4. Concern about burden on senior and family: The participants were hesitant to refer dependent older adults to dentists as they believed that visiting off-site dental clinics would be a significant physical and mental burden on them. In addition, they were concerned that family members would need to sacrifice their time and financial resources to accompany the older adult to off-site dental institutions.

Many of them have stiffness and contractures of their limbs and that is very difficult for them to go down to see a private dentist in the community. (MP05, Family physician, 39 y)

I struggle to see families who are disadvantaged, to see value in this, for them to pay for dental treatment. (MP07, Family physician with geriatric medicine diploma, 40 y)

### Enablers to Achieve Better Partnership with Dentists

Despite these barriers, participants also identified potential enablers for better collaboration with dental professionals at the end of their interview. They recognized that strengthening the partnership with dentists could benefit dependent older adults in light of holistic geriatric care.

It seems very clear now to me that they should have a dental person come in and see them regularly. . . . Yes, because it would make a difference to their lives in so many ways. (MP13, Geriatrician, 41 y)

#### Networking opportunity connecting dental and medical practitioners

Considering the traditional fragmentation of oral health care from other health care communities, participants emphasized that the networking pathway linking dental and medical areas would first need to be established to initiate a conversation around improving the oral health of older adults in LTCs.

Having a network or, you know, being able to tap on this kind of consultative corridor with the dentist, “Hey, you know, this guy has this. What will you do?” (MP03, Family physician, 39 y)

I believe that if we do look into how we can create that environment whereby there’s more collaboration, then I think that will be a starting point for us to collaborate more closely. (MP07, Family physician with geriatric medicine diploma, 40 y)

#### Opportunities for medical and geriatric care professionals to learn about oral health

Participants emphasized the importance of establishing formal and regular educational opportunities aimed at enhancing oral health awareness throughout the entire geriatric care community. This outreach should extend beyond medical students and practitioners to include other geriatric care professionals. They recognized the necessity of gaining a deeper understanding of oral health of dependent older adults in order to foster stronger partnerships with dentists. They were also particularly keen to learn practical knowledge on assessing dental issues, conducting initial oral management, and the range of services dentists can provide for dependent older adults. They believed that this knowledge and information would significantly enhance effective communication and collaboration with dental professionals.

The first thing that really needs to be done is I think we have to have the awareness first. . . . I would say that everyone needs a basic understanding so that there some awareness and some seed planted, but there needs to be a system in place. (MP13, Geriatrician, 41 y)

If we have more knowledge about the importance, I think it will give us more of a reason to go more in depth and to learn. (MP06, General practitioner, 34 y)

One of the participants shared her own experience where her awareness of oral health in the elderly significantly improved only after meeting a particular dentist.

Only when I got to know this particular dentist who really opened my eyes to this, I started to understand it (oral health) a lot better. . . . I saw that yes, there were a lot of oral health issues in the elderly. (MP13, Geriatricians, 41 y)

#### Establishing domiciliary dental service in the existing home care service framework

Since participants recognized that accessing off-site dental service was a major barrier for dependent older adults, they underscored the necessity of introducing a domiciliary dental service in existing home care service framework. Along with establishing such a domiciliary dental service, MPs addressed that dental subsidies also need to be expanded to facilitate easier access for dependent older adults and families to receive professional dental service.

If we can actually have like a small mobile dental clinic kind of setup that with portable equipment, hopefully that will help to let the dentist be able to do what they need to do, but also have something that is able to be located in our clinic—I mean the nursing home itself so that the residents don’t have to go out. (MP09, General practitioner with geriatric medicine and family medicine diploma, 39 y)

As long as there’s no subsidy for certain service, a lot of the elderly or the caregivers for the elderly are hesitant to take up the service, even if that service was available. (MP02, Family physician, 51 y)

## Discussion

This study provides crucial insights into how MPs involved in LTCs in Singapore viewed the importance of oral health care for dependent older adults. There was a clear recognition among participants that older adults in LTCs had significant oral health issues. However, this awareness did not result in their taking proactive steps that would lead to a proper oral health care intervention. This disconnect was driven by 3 primary barriers: (1) cultural beliefs that deprioritize oral health, (2) systemic challenges that discourage the use of oral health services, and (3) internal limitations that prevent MPs from making fully informed decisions in managing oral health issues. These factors collectively hinder effective collaboration between medical and dental professionals in addressing the oral health care needs of dependent older adults in LTC settings.

As highlighted in the introduction, oral health care in LTCs is often overlooked within the broader geriatric care agenda (Harnagea et al. 2017; Chávez et al. 2022), despite the call for greater interprofessional and holistic approach in LTCs (WHO Ageing and Health 2021). Our study reinforces this concern by demonstrating that although the participating MPs in Singapore were aware of the importance of oral health, its management falls outside their job scope in LTC settings. In addition, dental services are not well-integrated into the LTC system, creating a persistent disconnect between dental care and other aspects of geriatric health management. Through an in-depth exploration of MPs’ awareness, approaches, and the challenges they faced in collaborating with dental professionals, this study contributes new knowledge on the multifaceted barriers that hinder the seamless integration of oral health care into the broader geriatric care framework.

Despite the insightful findings of this study, we acknowledge its limitations. A key limitation is the potential challenge in transferring the findings to other contexts beyond Singapore. The health care system in Singapore, including its LTC settings, is highly specific, characterized by its unique blend of public and private health care services and its rapidly aging demographic. These factors contribute to a health care environment that may differ significantly from those in other countries, particularly in terms of resource allocation, cultural attitudes toward aging and health care, and dental services. However, despite these contextual specificities, the study’s findings may still offer valuable lessons for other countries, particularly those facing similar challenges with an aging population and the integration of oral health care in LTC settings. For example, the identification of barriers such as the fragmentation between medical and dental services, the lack of interdisciplinary collaboration, and the cultural undervaluation of oral health in geriatric care are issues that are not unique to Singapore (Bots-VantSpijker et al. 2014; Smith and Thomson 2017; Niesten et al. 2021). Notably, the only comparable study, conducted in Sweden in 2007, explored general MPs’ perceptions of oral health in older adults and identified barriers similar to those found in our study (Andersson et al. 2007), despite differing social and cultural contexts. These parallels suggest that this issue is not confined to Singapore but may represent a broader, systemic challenge across various health care settings. Countries facing similar health care challenges could apply our findings to gain insight into these complexities and explore potential solutions. Second, there may be volunteer bias as participants who have an interest in the oral health of older adults were more likely to participate in this study. However, this was reduced by including MPs with diverse perspectives, encompassing both those with high oral health awareness and those with low oral health awareness. To ensure a neutral and objective stance of the researchers during the interviews, researchers, who were unfamiliar with the current situation in the LTCs, were chosen as the main interviewers and analyzers. Following the independent initial data analysis conducted by multiple researchers, codes, themes, and interpretations were discussed multiple times until a consensus was reached. This process also helped to maintain the credibility of the findings. In addition, interviews were conducted virtually, which may be unconventional in qualitative studies. However, all participants of this study were already familiar with online meetings at the time and remained active in discussions. Furthermore, the option of turning off the video camera also encouraged them to be open and honest in their sharing.

In terms of cultural barriers, the age-based stereotype played a key role in the deprioritization of oral health care for older adults in LTCs in Singapore. Participants in this study noted that both families and eldercare professionals often assumed that oral health naturally deteriorates with age, leading to reduced efforts to improve or maintain oral function and seek professional dental interventions. This stereotype, which is widely recognized by the WHO as a global issue, often leads to the undervaluation of elderly patients’ health needs (Mikton et al. 2021). In the context of oral health, this bias manifests as the belief that poor dental health is an inevitable or normal part of aging, rather than a condition that can be treated and improved (Elliott et al. 2024). These attitudes reflect a broader societal tendency to accept the deterioration of health in older adults as unavoidable, influencing both systemic and internal barriers and further complicating the integration of comprehensive oral health care in LTC settings (Wyman et al. 2018). Addressing these cultural barriers will require a multifaceted approach. Public health campaigns and educational initiatives are essential to challenge such stereotypical beliefs, emphasizing that oral health should be maintained throughout life (Petersen and Ogawa 2018). Government-supported campaigns such as Australia’s “Healthy Mouths, Healthy Lives” (Australian Government Department of Health and Aged Care 2016) and Japan’s “8020 Campaign” (Ishii 2005) offer valuable models. In Singapore, one national dental institution has launched its own Oral Health Movement 8020 initiative (National Dental Centre Singapore 2022), targeting oral frailty in older adults. However, to effectively dispel this widespread misconception, scaling this initiative to a government-supported campaign would be a crucial next step. Enhancing geriatric-specific training in the eldercare community, as seen in these initiatives, would also empower health care providers to better advocate for the oral health needs of older adults.

While cultural barriers such as age-based stereotypes diminish the priority of oral health, systemic barriers further compound the issue by creating structural obstacles that limit access to care. The systemic barriers highlighted by MPs in this study reflect similar problems identified in focus group discussions with dentists and oral therapists in Singapore (Ho et al. 2023; Tada, Lee, Koh, et al. 2023; Koh et al. 2024). The alignment between the concerns raised by MPs and dentists in Singapore underscores the pervasive nature of underdeveloped integration of oral health care service in LTCs. Although this fragmentation is not unique to Singapore (Smith and Thomson 2017), there are some countries taking initiatives to enhance access to dental service such as Japan, Sweden, Belgium, Australia, and more. For example, Japan (Aida et al. 2021) and Sweden (Sjögren et al. 2010) have implemented integrated approaches to oral health care in LTCs. A common initiative in both countries is the use of mobile dental clinics to bring essential dental services directly to older adults in LTCs, leveraging the capabilities of dental hygienists for oral hygiene care, preventive care, and caregiver education and facilitating communication with interdisciplinary teams. This collaboration helps maintain oral health as a regular part of the overall eldercare regimen, addressing dental issues before they become severe. Furthermore, both Japan and Sweden provide financial support to make dental care more financially accessible for older adults. While international examples offer valuable insights, it is vital to acknowledge the financial implications when advocating for the integration of oral health care into policy making. The economic challenges faced by this population and the rising health care costs in aging societies are crucial issues that need to be addressed (McKenna et al. 2015). Looking forward, more research focused on cost-effective analysis will be crucial in evaluating the feasibility and sustainability of integrating similar models into LTCs. Understanding the financial implications and benefits of mobile dental clinics, enhanced training for oral therapist or care staff, and interdisciplinary collaboration will guide policy makers in adopting strategies that not only improve access to dental care but also optimize resource allocation.

Lastly, barriers within the medical community in Singapore further hindered effective collaboration with dental professionals. These challenges were closely linked to both cultural and systemic barriers and contributed to their reactive response to oral health issues. Given the current lack of accessible professional dental services for LTCs and the critical role of medical doctors in decision making regarding oral health issues, establishing a learning platform that bridges the gap between dental and MPs within the LTC system is essential as discussed by the participating MPs. Expanding opportunities in the existing continuing professional education course for all eldercare professionals to learn about geriatric oral health management would be the most feasible first step in Singapore. This initiative would not only enhance awareness of oral health but also promote a more comprehensive approach to the overall well-being of older adults in LTCs. Furthermore, to strengthen the partnership between medical and dental professionals, it is crucial to develop a service model that ensures all members of the eldercare team share a goal of oral health management. This can be achieved through the co-design of a comprehensive service framework that actively incorporates the perspectives and needs of all stakeholders, including MPs, dental professionals, caregivers, and the elderly themselves (Niesten et al. 2021). Such a collaborative approach will foster more coordinated and holistic care for older adults in LTCs.

## Conclusions

This study highlighted a significant gap in collaboration between MPs and dental professionals in Singapore, despite widespread awareness of the poor oral health status of care-dependent older adults in LTCs. Cultural, systematic, and internal barriers collectively contributed to the underdeveloped partnership between medical and dental professionals in LTCs. While these findings are specific to Singapore, they offer valuable insights that may be applicable to other countries facing similar challenges in integrating oral health care into LTCs. The results underscore the urgent need for policy and service transformations to strengthen collaboration and ensure comprehensive care for older adults. Future research should focus on exploring the long-term impact of integrated service models on both health outcomes and cost-effectiveness in LTCs, thereby guiding policy makers toward developing evidence-based, sustainable, and effective service models.

## Author Contributions

S. Tada, contributed to conception, design, data acquisition, analysis, and interpretation, drafted and critically revised the manuscript; S.M.Y. Koh, contributed to design, data acquisition, analysis, and interpretation, critically revised the manuscript; G.K.Y. Lee, contributed to data interpretation, critically revised the manuscript; M.L. Wong, contributed to design, critically revised the manuscript. All authors gave final approval and agree to be accountable for all aspects of the work.

## Supplemental Material

sj-docx-1-jct-10.1177_23800844241305015 – Supplemental material for Medical Practitioners’ Views on Barriers in Collaboration with Dentists in Long-Term Care Settings Supplemental material, sj-docx-1-jct-10.1177_23800844241305015 for Medical Practitioners’ Views on Barriers in Collaboration with Dentists in Long-Term Care Settings by S. Tada, S.M.Y. Koh, G.K.Y. Lee and M.L. Wong in JDR Clinical & Translational Research
