# Supplementary material for: Medical Practitioners’ Views on Barriers in Collaboration with Dentists in Long-Term Care Settings
Source: JDR Clin Trans Res. 2025 Feb 10;10(4):385–97. doi: 10.1177/23800844241305015 (PMC12402521; doi:10.1177/23800844241305015)
Supplement: sj-docx-1-jct-10.1177_23800844241305015 – Supplemental material for Medical Practitioners’ Views on Barriers in Collaboration with Dentists in Long-Term Care Settings [file sj-docx-1-jct-10.1177_23800844241305015.docx]

**Appendix (Table A1):**

| **Domain** | **Theme** | **Code** | **Definition** | **Quote Example** |
| --- | --- | --- | --- | --- |
| Awareness | General Awareness of Oral Health’s Impact on Overall Well-being | Awareness of Oral Health’s Impact on QoL | MPs knowing that oral health directly influences comfort, eating, and social interactions, thereby affecting the quality of life of older adults. | *if you have poor oral health and nutrition is not great. The other thing, of course, is image, you know, self-esteem and self-image as well… It can be that you can have difficulty articulating as well and speaking, which can also impact your social interactions…And then, of course, if you have bad oral health, it can also lead to halitosis, which can again affect interactions. And then and I would think that the rest of that is really more physical related, like discomfort and pain. [MP13]* |
|  |  | Awareness of Oral Health's Impact on General Health | MPs knowing that oral health is linked to systemic health, influencing conditions such as cardiovascular disease and diabetes. | *- Dental health itself also affects the outcome of pneumonia, right? [MP04] - I guess it'll contribute to their nutrition- malnutrition. [MP06]* |
|  |  | Awareness of Importance of Oral Hygiene Control | MPs knowing the role of regular oral hygiene in preventing dental problems and maintaining overall health | *I've been taught in medical school is sometimes oral health, oral hygiene can actually reduce the bacteria load [MP08]* |
|  | Recognition of Oral Health in Current LTC Contexts | Recognition of Poor Oral Health Status | MPs recognizing that older adults have poor oral hygiene / poor oral health status | *Poor oral health is quite common actually[MP02]* |
|  |  | Recognition of Need for Professional Dental Treatment | MPs recognizing that professional dental treatment is essential for addressing the complex oral health needs of older adults, beyond basic hygiene care | *we look into the mouth and they have poor hygiene, missing teeth, decayed teeth, loose teeth, dentures, but not wearing them because maybe they are loose or they have poor fitting dentures, which are causing sores. So we will then pick up all these issues and then we will then, of course, advice that they see a dentist. [MP07]* |
|  | Perceived Disconnect | Oral Health Viewed as Outside Medical Scope | MPs consider comprehensive oral health management to be beyond their primary responsibilities, focusing instead on issues that align more closely with their medical expertise. | *- So I think the nurses are already mostly doing it in the nursing homes. [MP05]*  *- But I would say that, unfortunately, this is not really our main focus. And so, yeah, so a lot of the times we look into the mouth really to look for like oral thrush, more of the medical aspect.[MP06]* |
| Approach | Proactive Response | Periodical Basic Oral Assessment | MPs routinely provides a brief screening of both dental and oral issues. | *“Basically, I see the number of teeth, I see the cleanliness of the teeth, whether there's any plaque or things like that, any ulcers, the moistness of the buccal cavity.” [MP09]* |
|  |  | Encourage or Educate Caregivers on Oral Hygiene Care | MPs educate and give advice to NH and family caregivers on how to improve oral care for older adults | *“so we just want to make sure that the caregivers are doing their regular oral toilet just to maintain the oral hygiene.” [MP12]* |
|  |  | Self-Study on Oral Health as they Encounter Oral Health Issues | MPs independently research and learn about oral health in response to specific issues they encounter with patients in clinical settings. | *I think a lot of it is self-reading...* *you’re forced to read up because you encounter problems in the ward. [MP02]* |
|  | Passive Response | Taking Actions Only When Complaints Arise | MPs tend to address oral health issues primarily in response to specific complaints from patients or caregivers or acute symptoms. | *when you see enough complaints being brought up by the primary caregiver, that’s* *you're forced to* *look into it. [MP02]* |
|  |  | Reprioritisation of Oral Health Issues | MPs tend to deprioritize oral health concerns in favor of other medical priorities. | *…getting access to denture services, to re-prescribe denture services is not at the top of the priority list very often. [MP04]* |
|  | Limited Professional Dental Intervention | Referral to Dentists Only for Acute or Severe Dental Issues | MPs only refer to dentist when the condition is severe such as acute pain, abscess, need for extraction, etc. | *“We usually only get dental intervention when there is something more drastic that happens, like, you know, a fracture tooth” [MP03]* |
|  |  | Symptom-Focused Care by Medical Doctors for Oral Health Issues | MPs prescribe anti-biotics/medication to control infection based on their own medical judgement or supplements to aid nutrition instead of treating/locating the root problem. | *- If you suspect some infection, we may give antiseptic mouthwash or oral antibiotics and then give some..[MP05]*  *- Unfortunately, I don't have any (dental) resources. I don't know any dentists actually, so a lot of times their diet will be downgraded. So softer.[MP08]* |
|  | Limited Collaboration with Dental Professionals | Consultation with Speech Therapist or Dietician for Eating Issues | MPs tend to refer older adults to speech therapist or nutritionist when they encounter eating or swallowing issues, rather then dentists. | *“in malnutrition, dentition is an important issue. Then, maybe I refer to a dietician and a speech therapist to select the appropriate type of food for them” [MP10]* |
|  |  | Limited Communication with Dental Professionals | MPs have never had any interaction or a little interaction with dentists regarding geriatric care. | *- I never communicated I think with a single dentist when I was working in the nursing home.” [MP13]*  *- I have not consulted with any dental health professionals. I mean, not directly regarding this because generally, there's no regular dental health professional attached to any of the nursing homes that I work at, even the one that works with a private clinic is kind of more of an ad-hoc basis. [MP09]* |
| Barrier | Cultural Barrier | Cultural Perception of Oral Health as Non-essential in Elderly Care | MPs feel that there is a belief, prevalent among families, healthcare professionals, and within the broader eldercare community, that oral health is of lower priority or less critical compared to other aspects of elderly care. | *- “Family members tend to just put it really low down the list. Yeah. And perhaps culturally it's also accepted that when you get older, yeah, you go down, you kind of downgrade your own diet.” [MP03]*  *- “All the aged care system emphasises on nursing, medical, physiotherapy, OT, ST and so on. But the dentist doesn't seem to be there.” [MP10]*  *- “I think even as a GP, I think a lot of our well patients or patients who are ambulatory, I feel that oral health is something that's not given enough emphasis.” [MP07]* |
|  | Internal Barrier | Lack of Educational Opportunities in Oral Health | MPs have little or no opportunity to learn about oral health in the medical academic program | *This (oral health) is not something that is taught very much in medical school [MP09]* |
|  |  | Knowledge Gaps and Confidence Issues in Oral Health Management | MPs experience the uncertainty and lack of confidence due to insufficient knowledge about when and how to involve dental professionals or manage oral health conditions | *Yeah, yeah. So is like you know, at which level, how.. I don't know.. what level of of poor dentition, poor oral hygiene should I get the dentists, you know, or whether just simple advice on oral care, oral hygiene will suffice [MP03]*  *I really have no idea on how to manage the dental conditions that many of them have. [MP05]* |
|  |  | Scepticism Toward Dental Interventions in Advanced Geriatric Cases | MPs have the doubts and uncertainties regarding the clinical benefits and outcomes of dental interventions for elderly patients with complex health conditions | *- I think what is lacking is I'm not sure.. what is.. how much additional benefit, you know, like which patient group will get the most benefit out of a consult with the dental professional? [MP03]*  *- I guess I'm not sure how much it really contributes clinically…I've been taught in medical school is sometimes oral hygiene management can actually reduce the bacteria load, but I'm not sure how this translates clinically [MP08]* |
|  |  | Concern about Burden on Senior and Family | MPs express apprehension about the physical, financial, and logistical challenges that accessing dental service poses for seniors with limited mobility and their families | *- “Many of them have stiffness and contractures of their limbs and that is very difficult for them to go to even go down to see a private dentist in the community.” [MP05]*  *-I struggle to see families who are disadvantaged, to see value in this, for them to pay for dental treatment. [MP04]*  *- “if they (family) are working that they find it quite hard to take leave sometimes to accompany the resident for the dental appointment. They want to save their leave for the more important things a cardiology appointment or things like that.” [MP09]* |
|  | Systemic Barrier | Systemic Fragmentation of Oral Health from Other Geriatric Areas | MPs feels that dental area is fragmented from geriatric care system which makes MPs feel dental issues outside of their purview. | *“I feel that the dental setup is always very separate from the medical setup… so it makes it harder for people to see it as being "oh, we are actually working towards the same thing", you know, in that sense.” [MP13]* |
|  |  | Inadequate Integration of Oral Healthcare in LTC Systems | MPs see the lack of structured and systematic approaches to incorporating oral health screening and professional care into the long-term care (LTC) systems for elderly populations. | *“there's no real structure in screening for dental health in this population. I mean, you have it for the children, you ave recommendations for adult population. You don't really have anything for elderly. You certainly don't have anything for nursing homes.” [MP04]* |
|  |  | Inaccessible Off-Site Dental Facilities for Seniors in LTCs | MPs experience difficulty accessing off-site dental care for LTC seniors due to uncertainties about dental clinics' ability to manage geriatric or bedbound patients. | *“because we also don't know what is the capability, some dentists, they may not be comfortable with managing geriatric oral health.” [MP07]*  *“I'm not sure if the normal dental clinics in the neighborhood will be able to handle bedbound patients.” [MP12]* |
|  |  | Logistical and Financial Barriers to Access | MP experience difficulties in accessing dental care for seniors in LTCs due to long waiting times and financial constraints, complicating timely dental treatment. | *And the polyclinics are really overwhelmed with routine general population dental health. And yeah, and the subsidy... subsidizing mechanism is different, I think. And so most times, you know, we would try them to the hospitals, which then is a wating time.[MP07]* |
